# A novel inverse membrane bioreactor for efficient bioconversion from methane gas to liquid methanol using a microbial gas-phase reaction

**DOI:** 10.1186/s13068-023-02267-6

**Published:** 2023-02-02

**Authors:** Yan-Yu Chen, Masahito Ishikawa, Katsutoshi Hori

**Affiliations:** Department of Biotechnology, Graduate School of Engineering, Nagoya University, Furo-Cho, Chikusa-Ku, Nagoya, 464-8603 Japan

**Keywords:** Gas-phase bioreaction, *Methylococcus capsulatus* (Bath), Methane, Methanol production, Membrane bioreactor

## Abstract

**Background:**

Methane (CH_4_), as one of the major energy sources, easily escapes from the supply chain into the atmosphere, because it exists in a gaseous state under ambient conditions. Compared to carbon dioxide (CO_2_), CH_4_ is 25 times more potent at trapping radiation; thus, the emission of CH_4_ to the atmosphere causes severe global warming and climate change. To mitigate CH_4_ emissions and utilize them effectively, the direct biological conversion of CH_4_ into liquid fuels, such as methanol (CH_3_OH), using methanotrophs is a promising strategy. However, supplying biocatalysts in an aqueous medium with CH_4_ involves high energy consumption due to vigorous agitation and/or bubbling, which is a serious concern in methanotrophic processes, because the aqueous phase causes a very large barrier to the delivery of slightly soluble gases.

**Results:**

An inverse membrane bioreactor (IMBR), which combines the advantages of gas-phase bioreactors and membrane bioreactors, was designed and constructed for the bioconversion of CH_4_ into CH_3_OH in this study. In contrast to the conventional membrane bioreactor with bacterial cells that are immersed in an aqueous phase, the filtered cells were placed to face a gas phase in the IMBR to supply CH_4_ directly from the gas phase to bacterial cells. *Methylococcus capsulatus* (Bath), a representative methanotroph, was used to demonstrate the bioconversion of CH_4_ to CH_3_OH in the IMBR. Cyclopropanol was supplied from the aqueous phase as a selective inhibitor of methanol dehydrogenase, preventing further CH_3_OH oxidation. Sodium formate was added as an electron donor to generate NADH, which is necessary for CH_3_OH production. After optimizing the inlet concentration of CH_4_, the mass of cells, the cyclopropanol concentration, and the gas flow rate, continuous CH_3_OH production can be achieved over 72 h with productivity at 0.88 mmol L^−1^ h^−1^ in the IMBR, achieving a longer operation period and higher productivity than those using other types of membrane bioreactors reported in the literature.

**Conclusions:**

The IMBR can facilitate the development of gas-to-liquid (GTL) technologies via microbial processes, allowing highly efficient mass transfer of substrates from the gas phase to microbial cells in the gas phase and having the supplement of soluble chemicals convenient.

**Supplementary Information:**

The online version contains supplementary material available at 10.1186/s13068-023-02267-6.

## Background

Methane (CH_4_), the main component of biogas, shale gas, and gas hydrate, has been used as the major energy resource for humans. After the shale gas revolution in the United States, CH_4_ became a popular chemical feedstock due to its high availability. However, because CH_4_ exists in a gaseous state in ambient conditions, large amounts of CH_4_ dissipation occur through the entire gas supply chain, resulting in severe greenhouse effects [1]. Of the various candidate technologies for effectively utilizing CH_4_, liquefied natural gas (LNG) is a common form to transport CH_4_ from its production site to distant consumption sites. However, the cost is quite expensive due to the extremely low temperature needed for storage and transportation. Converting CH_4_ into methanol (CH_3_OH) through a gas-to-liquid (GTL) technique has attracted much attention because of the ease of storage and transportation of liquid CH_3_OH [2, 3]. CH_3_OH is a useful feedstock for further chemical synthesis, a fuel with high energy density, and an excellent hydrogen carrier that provides safe and clean energy. However, the chemical process of producing CH_3_OH through direct functionalization of CH_4_ is energy intensive (the reaction temperature is approximately 900 °C and the operation pressure is 0.5–4 MPa) due to the high energy to cleave the C–H bond of CH_4_ (104 kcal mol^−1^) [4].

The use of methanotrophs, which are methane-oxidizing microorganisms, as whole-cell catalysts has attracted great attention, because methanotrophs can biologically convert CH_4_ to CH_3_OH by their methane monooxygenase (MMO) under ambient conditions in a single step [5–7]. Although CH_4_ bioconversion shows great potential as an economical GTL technology, it still faces some difficulties in achieving efficient conversion for commercialization. One of the difficulties occurs due to the low solubility of CH_4_ and oxygen (O_2_) in water. Since the conventional methanotrophic process is generally conducted in the aqueous phase [8–13], the limitation of the amount of dissolved CH_4_ results in a low productivity of CH_3_OH. Although various configurations of bioreactors for methanotrophic reactions have been proposed to enhance the process of dissolving CH_4_ in the aqueous phase, such as stirred tank reactors and bubble-lift reactors [14–16], the process consumes high amounts of energy to deliver gaseous substrates to the cells by stirring or bubbling.

Gas-phase bioreactions have been proposed to attain highly efficient delivery of gaseous substrates from the gas phase to whole-cell catalysts without stirring or bubbling [17, 18]. In gas-phase bioreactors, it is necessary to immobilize microbial cells on solid supports instead of dispersing them in an aqueous phase. A very large barrier of mass transfer of gaseous substrates from the gas phase to the aqueous phase is largely reduced or disappears in the absence of the bulk liquid. In the case of methanotrophs, cells immobilized on polypropylene rings or porous building materials have been used for CH_4_ degradation in the gas phase [19–21]. Some studies have claimed that microbial cells encapsulated in hydrogels can be used for gas-phase bioreactions, such as trichloroethylene degradation by *Methylocystis* sp. M (strain M) encapsulated in hydrogel beads [22]. However, we believe that systems using gels that contain a high content of water to encapsulate cells should not be recognized as gas-phase reactions. In fact, our previous study showed that the degradation rate of CH_4_ by *Methylococcus capsulatus* (Bath) cells encapsulated in alginate gel was as slow as that in a static aqueous phase [23].

In contrast to the degradation of gaseous substrates, there are very few reports on GTL bioproduction in the gas phase; the research is limited to the production of highly volatile chemicals, which are easily harvested from the gas phase. For example, Hou produced propylene oxide from propylene using *Methylosinus* sp. CRL 31 immobilized on porous glass beads in a gas‒solid bioreactor [24]. In the case of CH_3_OH, which has a hydrophilic liquid form at normal pressure and ordinary temperature in most bioreactions, it is difficult to harvest the gaseous product unless vaporization is enhanced by heating and/or decompression. In addition, an inhibitor for methanol dehydrogenase (MDH) and an electron donor, such as sodium formate, are usually needed for CH_3_OH production by wild-type methanotrophs [9, 25, 26], but supplying these chemicals in gas-phase bioreactors is quite difficult.

Membrane bioreactors (MBRs), which combine bioprocesses with membrane filtration, are popular wastewater treatment systems, because they realize simultaneous biological reactions and solid‒liquid separations. Recently, membrane aerated reactors, in which membranes are used for air supply rather than for solid‒liquid separation, have attracted attention [27, 28]. In these types of MBRs, O_2_ and/or gaseous substrates are supplied from the gas phase, transported through the membrane, and delivered to microbial cells in the aqueous phase. The microbial cells are suspended in the aqueous phase or immobilized on the membrane as a biofilm and catalyze the oxidation of organic chemicals in the aqueous phase. These MBRs have been employed for aerobic wastewater treatment [29, 30], synthesis of fine chemicals [31, 32], and CH_3_OH production [33, 34], in which CH_4_ and O_2_ were supplied through two independent membrane modules; premixing was not performed to keep the risk of explosion low. However, the disadvantage of membrane aerated reactors is the requirement of gas pressurizing to assist gas diffusion through the membrane into the aqueous phase, which is energy-consuming. Because of the development of biofilms on the membrane on the side of the aqueous phase, the transmembrane pressure must be increased over time to maintain the gas permeability.

The purpose of this study was to develop a new energy-efficient gas-phase bioreactor for bioconversion from CH_4_ gas into liquid CH_3_OH. A novel idea for a new bioreactor is to place filtered methanotroph cells on a membrane filter in the gas phase, which is a combination of a gas-phase bioreactor and an MBR. The usefulness of this new bioreactor, an inverse membrane bioreactor (IMBR), was demonstrated for GTL.

## Results

### Bioconversion of methane to methanol in a conventional membrane bioreactor

**Fig. 1:**
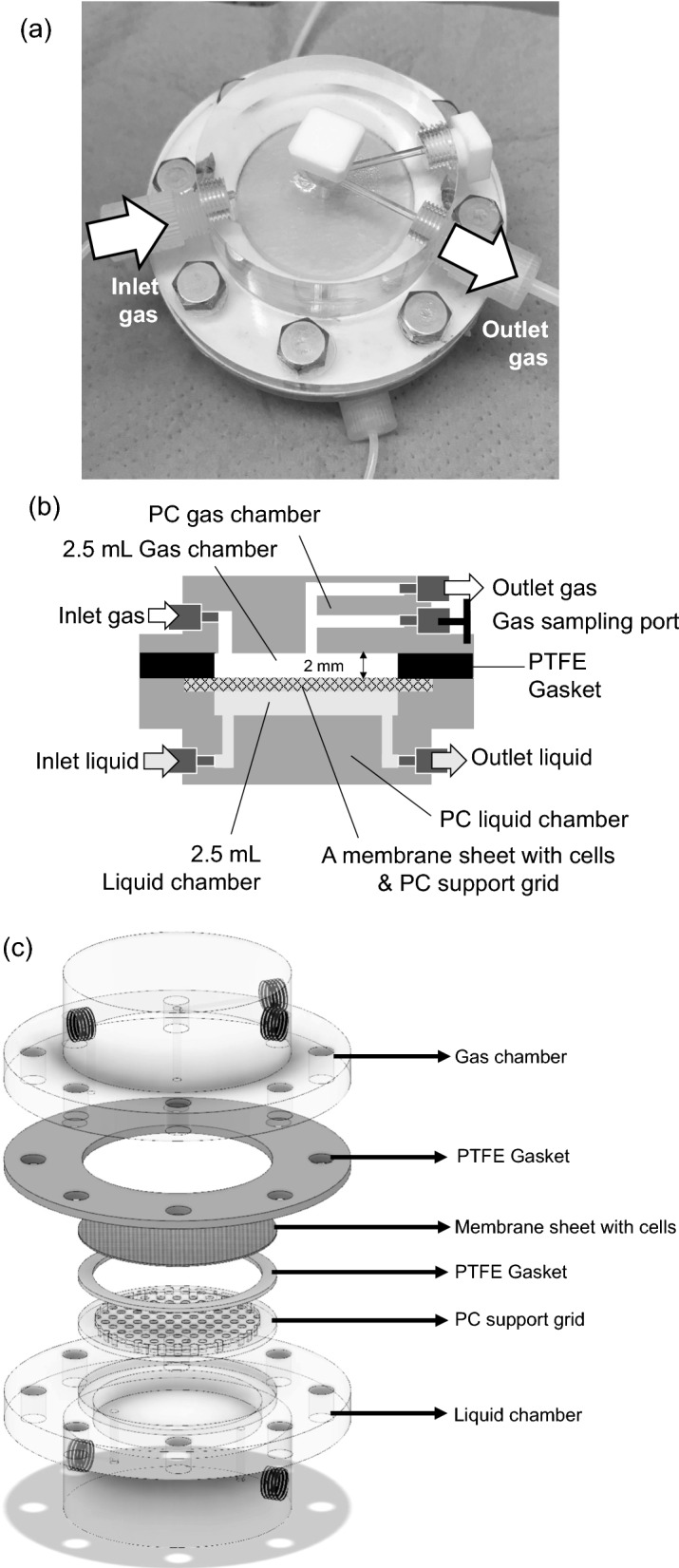
**a** Photo of the bioreactor designed and fabricated in this study. **b** Schematics of the reactor design. **c** Components for assembling the reactor

**Fig. 2:**
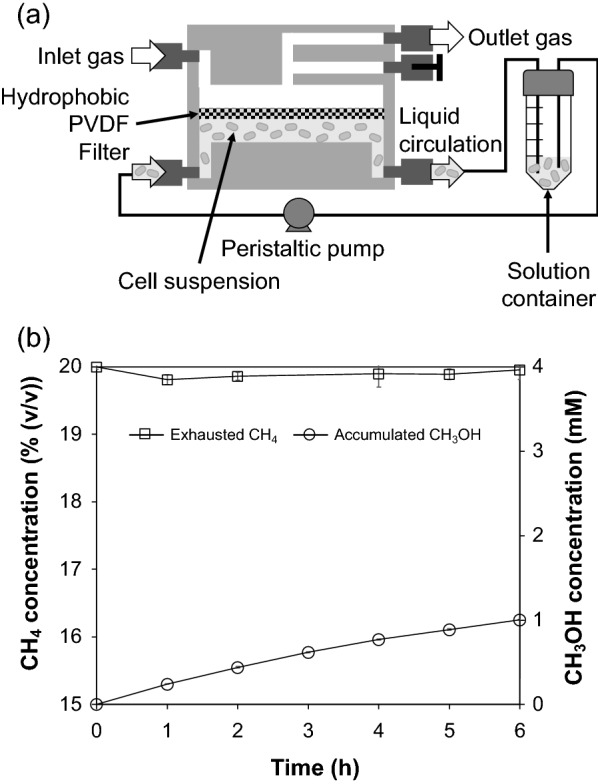
**a** Schematic of the operation of a conventional membrane bioreactor (MBR) assembled with a hydrophobic PVDF filter. **b** Time courses of CH_4_ concentration in the exhausted gas and the accumulated concentration of CH_3_OH in the solution container in the conventional MBR. The gas phase and the aqueous phase in the bioreactor were conducted under continuous operation and batch operation, respectively

We constructed a new MBR (Fig. 1a). Its detailed structure and assembly drawing are shown in Fig. 1b, c, respectively. A sheet of flat membrane on a support grid separates two chambers for the gas phase and the aqueous phase. First, this reactor was used for the bioconversion of CH_4_ into CH_3_OH as a conventional MBR, in which a hydrophobic polyvinylidene difluoride (PVDF) filter was employed to efficiently transport CH_4_ to the bottom aqueous phase. A cake of *M. capsulatus* (Bath) cells of 12.5 mg-dry cell weight (DCW) on the PVDF filter, which was prepared by filtration, was set in the middle of the reactor, facing the aqueous phase. However, the filtered cells that were immersed in the aqueous phase were detached, and the cells were resuspended in the aqueous solution that contained 10 μM cyclopropanol as an MDH inhibitor and 10 mM sodium formate as an electron donor. The released cells were circulated at 10 mL min^−1^ in the bottom liquid chamber that contained 10 mL of the aqueous solution (Fig. 2a). The mixed gas containing 20% (*v*/*v*) CH_4_ in air was continuously infused into the top gas chamber from the inlet at 3 mL min^−1^ of the gas flow rate without gas pressurization. The time courses of the CH_4_ concentration of the exhausted gas and the accumulated concentration of CH_3_OH in the solution container are shown in Fig. 2b. During the 6 h reaction, the concentration of CH_4_ at the outlet was maintained at 19.9% (*v*/*v*). By calculating the difference between CH_4_ concentrations at the inlet and the outlet, we estimated that the average consumption rate of CH_4_ was 7.1 μmol h^−1^. CH_3_OH produced by the circulating *M. capsulatus* cells accumulated in the liquid chamber, and its concentration gradually increased to 1 mM in 6 h. As a result, the conventional MBR without gas pressurizing had a low consumption ratio of CH_4_ (0.5%) and an average production rate of CH_3_OH (1.7 μmol h^−1^).

### Bioconversion of methane to methanol in an inverse membrane bioreactor

**Fig. 3:**
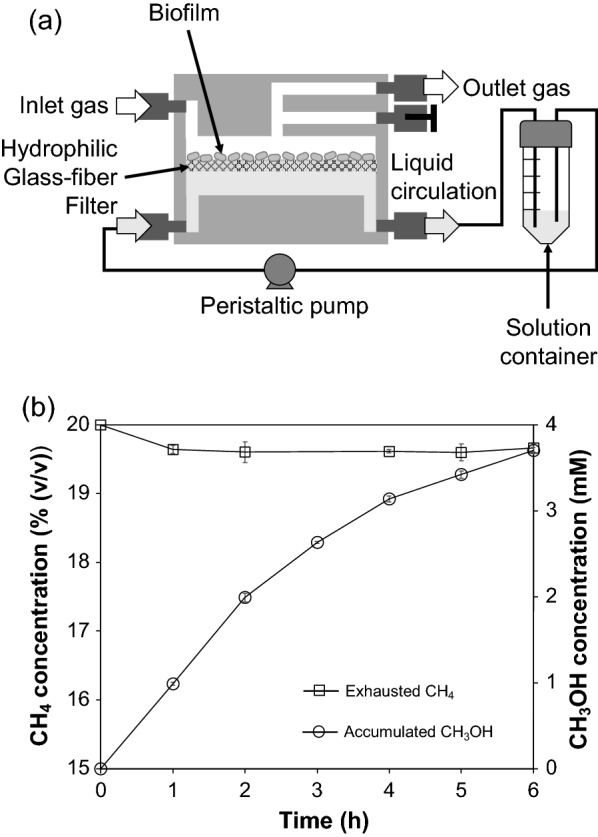
**a** Schematic of the operation of an inverse membrane bioreactor (IMBR), which was assembled with a hydrophilic glass–fiber filter. **b** Time courses of CH_4_ concentration in the exhausted gas and the accumulated concentration of CH_3_OH in the solution container in the IMBR. The gas phase and the aqueous phase in the bioreactor were conducted under continuous operation and batch operation, respectively

To simultaneously deliver gaseous CH_4_ to the methanotrophic cells, supply cyclopropanol and formate from the aqueous phase, and harvest CH_3_OH from the aqueous phase, we developed a novel MBR, IMBR, which had the same reactor configuration as described above; however, the membrane sheet was placed in an inverse direction to that of the conventional MBRs, so that the filtered cells were faced the gas phase (Fig. 3a). *M. capsulatus* (Bath) cells were filtered on a sheet of hydrophilic glass fiber filter, which was employed to efficiently transport water and soluble chemicals through the membrane and was set, so that the cells were not immersed in the aqueous solution in the liquid chamber. Thus, gaseous CH_4_ and O_2_ were directly delivered to the filtered whole-cell catalysts in the gas phase, and the produced CH_3_OH was transported to the aqueous phase via the hydrophilic membrane. In contrast, chemicals in the aqueous phase, including cyclopropanol, sodium formate, and inorganic nutrients, were delivered to the filtered cells from the aqueous phase via the membrane. A peristaltic pump was used for liquid circulation and to harvest CH_3_OH from the solution container. The water level in the solution container remained lower than the position of the membrane sheet in the IMBR, generating negative pressure in the direction from the filtered cells to the liquid chamber. Thus, the cake of the filtered cells was maintained in a semidry condition on the hydrophilic membrane in the gas phase.

Using this new IMBR, bioconversion of CH_4_ to CH_3_OH was performed. All the operation parameters were the same as those in the conventional MBR except the direction of the filtered cells and the material of the membrane filter. The time courses of the CH_4_ concentration of the exhausted gas at the outlet and the concentration of CH_3_OH accumulated in the solution container are shown in Fig. 3b. After infusing CH_4_ into the IMBR, the concentration of CH_4_ decreased from 20% (*v*/*v*) at the inlet to 19.6% (*v*/*v*) at the outlet. CH_3_OH was rapidly produced and accumulated in the aqueous phase; the concentration of accumulated CH_3_OH reached 2.0 mM in 2 h and then gradually increased to 3.7 mM in 6 h, which was approximately 4 times higher than that with the conventional MBR. The overall CH_3_OH productivity was 0.62 mmol L^−1^ h^−1^ in the IMBR with a batch operation of the aqueous phase. The formate concentration, which supplies reducing power for CH_4_ oxidation, decreased from 10 to 3.8 mM in 6 h (Additional file 1). The value of pH increased from 7.0 to 8.3, likely due to the consumption of proton produced by formate conversion into CO_2_ during CH_3_OH production. The OD values of the aqueous solution remained below 0.005 in 6 h (Additional file 2), showing no significant detachment of cells during the experiment. During the 6 h operation, the average consumption rate of CH_4_ was 25.3 μmol h^−1^, which was significantly enhanced in the IMBR compared with the conventional MBR operated as a negative control (Fig. 2), while the overall conversion of CH_4_ to CH_3_OH was 24.4% and 23.5% in the IMBR and the conventional MBR, respectively. This indicates that even though the cell activities in these two systems are almost identical, resistance to the mass transfer of CH_4_ limits the rate of CH_4_ consumption and dominates the rate of CH_3_OH production. Thus, the gas-phase bioreaction in the IMBR increased the rate of CH_4_ consumption compared with that of the aqueous-phase bioreaction and caused an increase in the rate of CH_3_OH production.

### Effects of the operating conditions of the IMBR on the consumption and conversion of methane

**Fig. 4:**
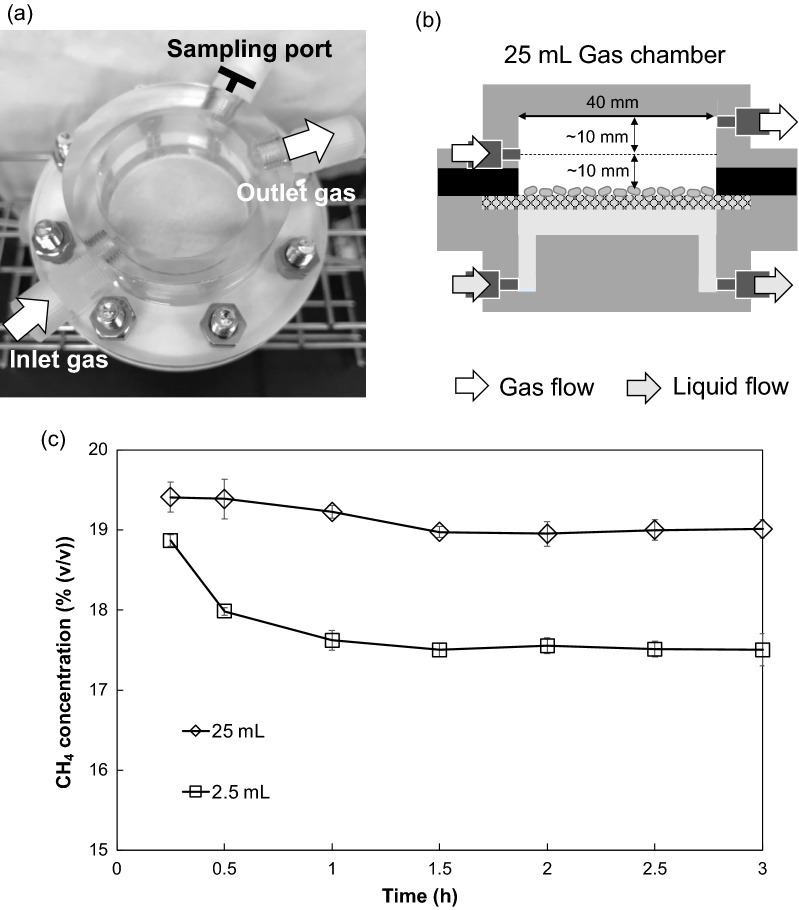
**a** Photo of the bioreactor with a 25 mL gas chamber. **b** Schematics of the IMBR with a 25 mL chamber. **c** Time courses of the concentration of exhausted CH_4_ in the IMBR with two sizes of the gas chamber, 2.5 mL and 25 mL

In the aforementioned result (Fig. 3b), the consumption ratio of CH_4_, which directly demonstrates the efficiency of the substrate utilization, was very low (only 2%). The space time, which is defined as the mean residence time of reactants in the reactor, is determined by calculating the ratio between the gas chamber volume and the volumetric flow rate of the inlet gas. In a well-mixed condition in a bioreactor, in which reactants and biocatalysts efficiently collide by agitation or bubbling for the liquid phase (such as an activated sludge process), the space time is close to the actual residence time of reactants, and therefore, a longer space time results in a higher conversion or consumption ratio. In our IMBR, although the gas phase was not agitated, the diffusion rate of gaseous reactants with a small molecular mass, such as CH_4,_ was quick enough in the gas phase that gaseous reactants were expected to contact the filtered cells efficiently. Therefore, the increase in the space time might improve the ratio of methane consumption. To confirm this, a new reactor with a gas chamber volume of 25 mL (Fig. 4a, b) was fabricated. In this reactor, the outlet was also repositioned on the opposite side of the inlet, as far away from the inlet as possible, to prevent the gas from passing through without contacting the filtered cells. This IMBR with the large gas chamber was compared with the previously mentioned IMBR with the smaller gas chamber in terms of the consumption ratio of CH_4_. The glass fiber filter with 10 mg-DCW of filtered *M. capsulatus* (Bath) cells was set between the gas and liquid chambers of these IMBRs, and the aqueous solution in the absence of cyclopropanol and sodium formate was infused into the liquid chamber and circulated at 10 mL min^−1^. The volumetric flow rate of the inlet gas containing 20% (*v*/*v*) CH_4_ was fixed at 1 mL min^−1^.

As shown in Fig. 4c, in the IMBR with the larger gas chamber, the CH_4_ concentration at the outlet decreased to 19.0% (*v/v*) during the first 1.5 h and remained constant afterward. When the IMBR with the smaller gas chamber was used, the exhausted CH_4_ concentration decreased sharply over time, reaching 17.6% (*v/v*) after 1 h, and then became constant at approximately 17.5% (*v/v*). In the gas chambers of 2.5 mL and 25 mL, the space time of the inlet gas was 2.5 min and 25 min, respectively. Although the space time in the gas chamber of 25 mL was 10 times longer than that in the gas chamber of 2.5 mL, the consumption ratio of CH_4_ was much lower (approximately 1/25) in the larger gas chamber than that in the smaller one (Additional file 3). Therefore, other operating conditions had to be examined to improve the consumption ratio of CH_4_, and the IMBR with a 2.5 mL gas chamber was used in further experiments.

**Fig. 5:**
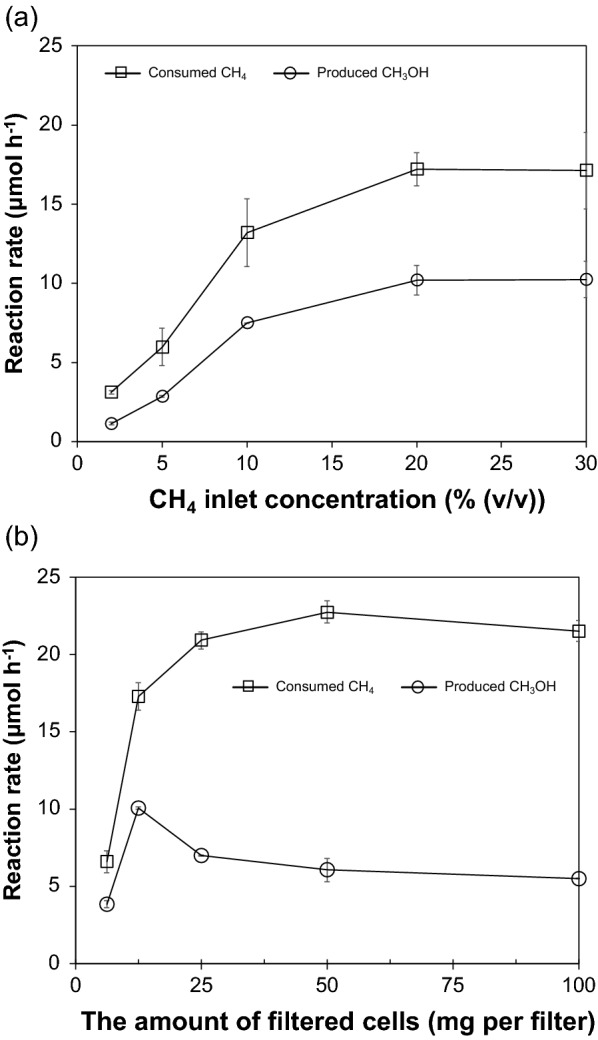
Effects of the inlet concentration of CH_4_ **a** and the filtered cell amount **b** on the consumption rate of CH_4_ and the production rate of CH_3_OH

Next, we examined the effects of the CH_4_ concentration of the inlet gas on the CH_4_ consumption and CH_3_OH production in the IMBR. For this purpose, *M. capsulatus* (Bath) cells of 12.5 mg-DCW on the membrane were faced the gas phase in the IMBR, in which the bottom liquid chamber carried 10 mL of the aqueous solution containing 10 μM cyclopropanol and 10 mM sodium formate, with circulation at 10 mL min^−1^. A gas containing CH_4_ at concentrations from 2% to 30% (*v*/*v*) in air was continuously pumped into the gas chamber at a gas flow rate of 1 mL min^−1^. The consumption rate of CH_4_ and the production rate of CH_3_OH were determined by the total amounts of CH_4_ consumed and CH_3_OH produced in the first 1 h of the reaction. Thereafter, from the ratio of these two values, the conversion of CH_4_ into CH_3_OH as a percentage was calculated. As a result, the CH_4_ consumption rate increased from 3.1 to 17 μmol h^−1^, and the consumption ratio of CH_4_ increased from 0.7% to 3.7% when the CH_4_ concentration of the inlet gas increased from 2% to 20% (*v*/*v*), but both of the values remained constant when the CH_4_ concentration was higher than 20% (*v*/*v*) (Fig. 5a and Additional file 4a). The CH_3_OH production rate showed the same trend as the CH_4_ consumption rate; it increased from 1.1 to 10 μmol h^−1^ on 2–20% (*v/v*) CH_4_ and remained constant at > 20% (*v/v*) CH_4_. This suggested that CH_3_OH production was dominated by MMO activity when MDH activity was inhibited and excess NADH was supplemented by the addition of sodium formate. The conversion of CH_4_ into CH_3_OH increased from 37% to 60% when the CH_4_ concentration in the inlet gas increases up to 20% (*v/v*) (Additional file 4a). Therefore, the optimal CH_4_ concentration was 20% (*v/v*) under the operating conditions of the IMBR.

We also examined the effect of the mass of filtered *M. capsulatus* (Bath) cells on the CH_4_ consumption and CH_3_OH production in the IMBR. For this purpose, *M. capsulatus* (Bath) cells from 6.25 to 100 mg-DCW on the membrane were faced the gas phase in the IMBR, which was run in the same manner as above except that the CH_4_ concentration in the inlet gas was fixed at 20% (*v*/*v*). The CH_4_ consumption rate and consumption ratio increased from 6.6 to 23 μmol h^−1^ and from 1.4% to 4.9%, respectively, as the mass of filtered cells increased from 6.25 to 50 mg-DCW (Fig. 5b and Additional file 4b). On the other hand, the CH_3_OH production rate increased from 3.8 μmol h^−1^ at 6.25 mg-DCW to 10 μmol h^−1^ at 12.5 mg-DCW but decreased to 7.0 μmol h^−1^ at 25 mg-DCW and to 6.1 μmol h^−1^ at 50 mg-DCW. Consequently, the conversion from CH_4_ to CH_3_OH was approximately 60% when the mass of filtered cells increased from 6.25 to 12.5 mg-DCW but decreased to 27% when the mass increased to 50 mg-DCW. Therefore, the mass of the cells is the optimum at 12.5 mg-DCW under the operating conditions for the IMBR.

**Fig. 6:**
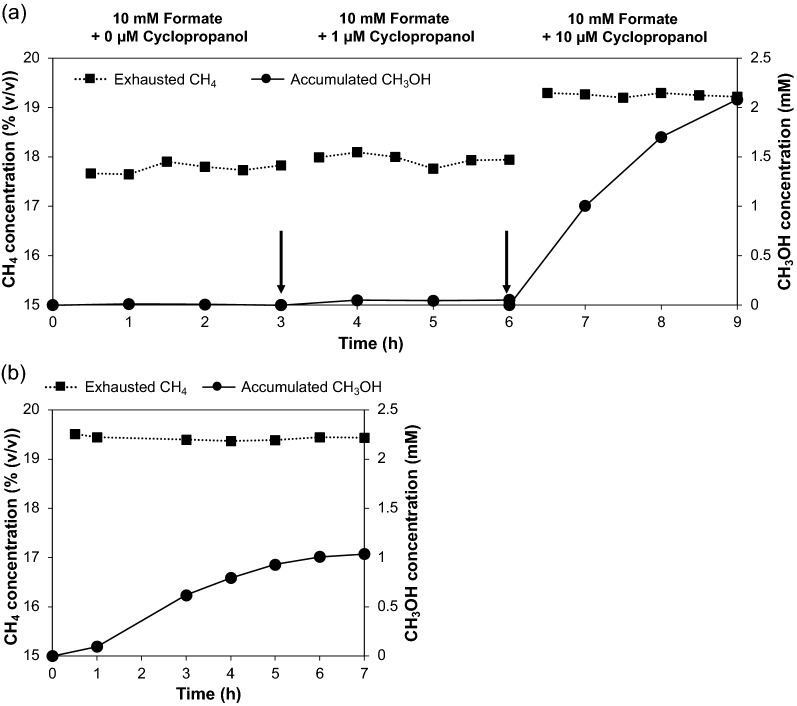
**a** Effects of the cyclopropanol concentration from 0 to 10 μM on the concentrations of exhausted CH_4_ and accumulated CH_3_OH. The arrows indicate the timing of the exchange of the aqueous solution. **b** Time courses of the concentrations of exhausted CH_4_ and accumulated CH_3_OH with 20 μM cyclopropanol

Moreover, we investigated the effect of the concentration of cyclopropanol in the aqueous solution on the CH_4_ consumption and CH_3_OH production in the IMBR. *M. capsulatus* (Bath) cells at 12.5 mg-DCW on the membrane were faced the gas phase in the IMBR. The inlet gas containing 20% (*v*/*v*) CH_4_ in air was pumped at 1 mL min^−1^ into the reactor. The aqueous solution was infused into the bottom chamber and circulated at 10 mL min^−1^. The solution was exchanged successively every 3 h by the solution containing the same concentration of sodium formate at 10 mM but different concentrations of cyclopropanol at 0 μM, 1 μM and 10 μM. Figure 6a shows the concentrations of exhausted CH_4_ and accumulated CH_3_OH in three periods with three different concentrations of cyclopropanol. In the first 3 h of operation, CH_3_OH was not produced because of the absence of cyclopropanol, and the exhausted CH_4_ concentration remained at approximately 17.8% (*v/v*). During the period with 1 μM cyclopropanol, a small amount of CH_3_OH was produced. However, the conversion of CH_4_ into CH_3_OH was not sustained and decreased from 4.6% to 1.6% (Additional file 5). The exhausted CH_4_ concentration slightly increased to 18.0% (*v/v*) in this period. At 6 h, the solution was exchanged again, and the concentration of cyclopropanol was increased to 10 μM. During this period, although the exhausted CH_4_ concentration increased to 19.3% (*v/v*), CH_3_OH was significantly accumulated and reached over 2 mM. The conversion was initially approximately 60% at 7 h and then decreased to 21% at 9 h. With the same conditions as above except for the aqueous phase, which contained 20 μM cyclopropanol, its effects on the concentrations of exhausted CH_4_ and accumulated CH_3_OH are shown in Fig. 6b. A higher concentration of cyclopropanol was expected to greatly inhibit MDH activity to produce more CH_3_OH. However, the maximum accumulated CH_3_OH concentration reached only 1 mM in 7 h, and the exhausted CH_4_ kept about 19.4% (*v/v*), while the maximum conversion decreased to approximately 20% by the increase in the cyclopropanol concentration (Additional file 6). Thus, the optimal cyclopropanol concentration was 10 μM under the operating conditions of the IMBR. However, at all the cyclopropanol concentrations tested, the conversion of CH_4_ into CH_3_OH decreased with time, suggesting that the continuous supply of cyclopropanol is necessary to maintain the conversion.

### Continuous bioconversion of methane into methanol in an IMBR

**Fig. 7:**
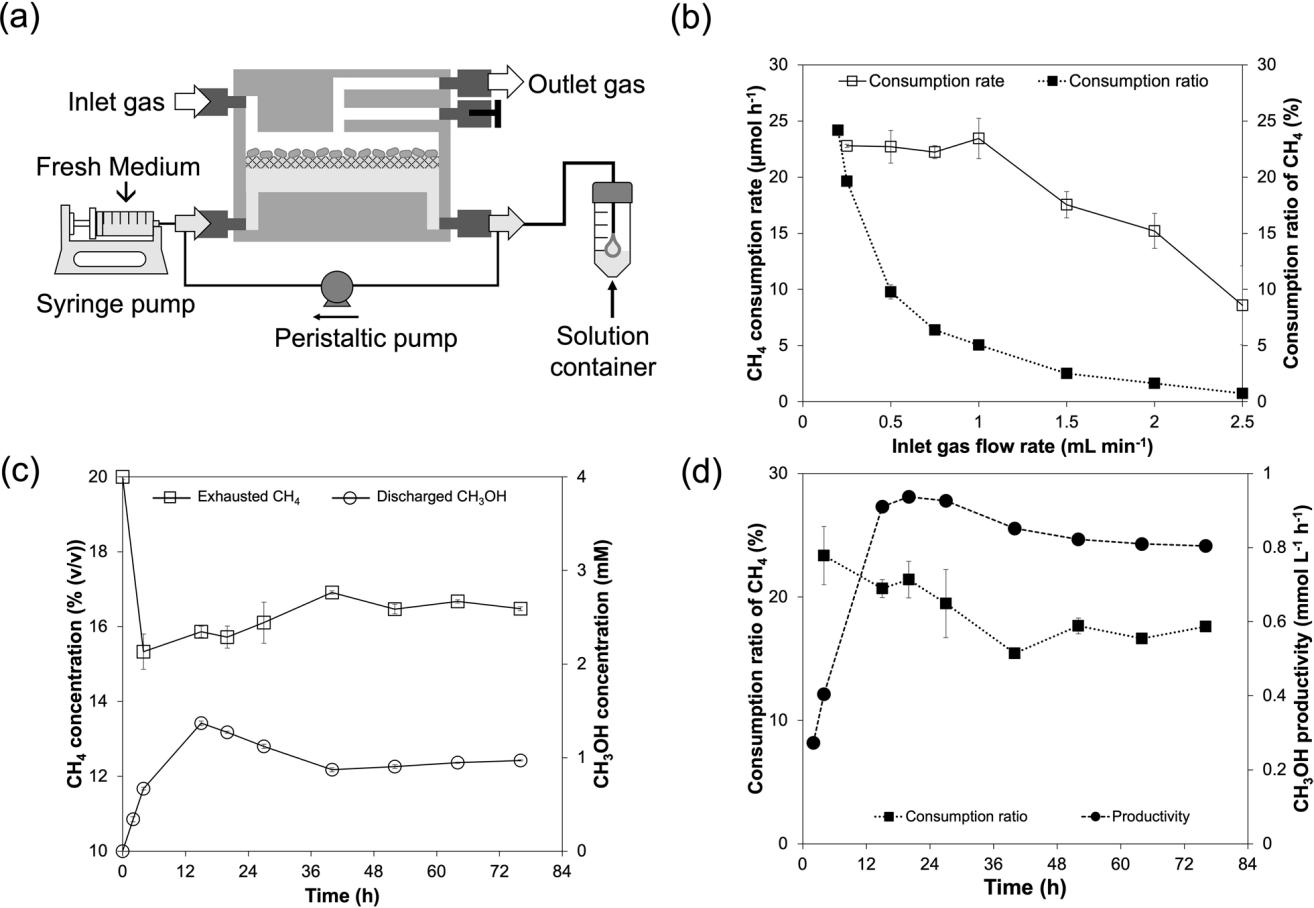
**a** Schematic of the continuous operation for the bioconversion of CH_4_ to CH_3_OH in the IMBR. **b** Effects of the gas flow rate on the consumption rate and consumption ratio of CH_4_. **c** Time courses of concentrations of CH_4_ and CH_3_OH at the outlet of the IMBR at 0.2 mL min^−1^ of the gas flow rate and 4 mL h^−1^ of the liquid flow rate. **d** Time courses of the consumption ratio of CH_4_ and CH_3_OH productivity in the IMBR

Finally, we carried out the continuous production of CH_3_OH from CH_4_ in a gas phase using the IMBR in the operating conditions that were optimized above (Fig. 7a). A mixed gas containing 20% (*v*/*v*) CH_4_ and air was continuously supplied into a 2.5-mL gas chamber of the IMBR, in which *M. capsulatus* (Bath) cells of 12.5 mg-DCW on the glass fiber filter were faced the gas phase, and the same volume of supplied gas was exhausted from the gas chamber. A total volume of 10 mL of the medium containing 10 μM cyclopropanol and 10 mM sodium formate, as well as 9.9 mM nitrate as a nitrogen source, was circulated through a 2.5 mL liquid chamber of the IMBR at 10 mL min^−1^ using a peristaltic pump. To replenish formate and other medium components that were consumed and to supply active cyclopropanol, in this experiment, the fresh medium was continuously injected into the liquid chamber at 4 mL h^−1^ of the flow rate using a syringe pump, and the same volumetric flow of the liquid as that injected was discharged into a solution container. The space velocity for the fresh medium was 1.6 h^−1^.

First, the gas flow rate was varied from 0.2 to 2.5 mL min^−1^ to investigate its effect on the reaction. Figure 7b shows the effect of the gas flow rate on the CH_4_ consumption rate and the consumption ratio of CH_4_ in the initial 1 h of the reaction. The CH_4_ consumption rate in the IMBR remained constant at approximately 23 μmol h^−1^ when the gas flow rate was in the range from 0.2 to 1 mL min^−1^, while the consumption ratio of CH_4_ decreased from 24.2% to 5.1% in this range. On the other hand, the CH_4_ consumption rate decreased from 23 to 8.6 μmol h^−1^ when the gas flow rate increased from 1 to 2.5 mL min^−1^, and the consumption ratio of CH_4_ further decreased to 0.7%. This suggests that the space time of CH_4_ in the IMBR is too short to maintain the same CH_4_ consumption rate as that at 1 mL min^−1^ when the gas flow rate is above 1.5 mL min^−1^. Then, we tried to run the IMBR by flowing the gas at 0.2 mL min^−1^ in its gas chamber for more than 3 d for the continuous bioconversion of CH_4_ into CH_3_OH by a microbial gas-phase reaction.

The time courses of CH_4_ concentration in a gas exhausted from the gas chamber and CH_3_OH concentration in a liquid discharged from the liquid chamber are shown in Fig. 7c. The CH_4_ concentration in the exhausted gas decreased from 20.0% to 15.3% (*v*/*v*) in the first 4 h of the reaction, increased slightly to 16.9% (*v*/*v*) in the next 36 h, and thereafter remained at approximately 16.7% (*v*/*v*) in the steady state. The variation in the CH_3_OH concentration of the discharged solution showed a trend in response to that of the exhausted CH_4_ concentration, although the response was slightly delayed. The produced CH_3_OH initially increased and reached 1.4 mM in 15 h. Then, it slowly decreased along with the drop in the consumption rate of CH_4_ and then remained constant at 1.0 mM after 40 h to the end of the experiment. The CH_4_ consumption ratio and CH_3_OH productivity over time are shown in Fig. 7d. Before 36 h, the consumption ratio of CH_4_ decreased from 23.4% to 17.6% but kept the ratio during the steady state. CH_3_OH productivities increased from 0.27 mmol L^−1^ h^−1^ at 2 h to 0.94 mmol L^−1^ h^−1^ (the maximum) at 20 h, and kept over 0.8 mmol L^−1^ h^−1^ (0.88 mmol L^−1^ h^−1^ in average) during the steady state. The conversion at each timepoint, calculated from the ratio of the CH_3_OH production rate to the CH_4_ consumption rate, is summarized in Table 1. The maximum conversion was 37% at 15 h. The average conversion in the steady state was approximately 27%, the average value of those at 52 h, 64 h, and 76 h in Table 1.

**Table 1:** CH_4_ consumption rate, the CH_3_OH production rate, and the conversion of CH_4_ to CH_3_OH in the continuous operation shown in Fig. 7

Time (h)	CH_3_OH production rate (μmol h^−1^)	CH_4_ consumption rate (μmol h^−1^)	Conversion (%)
4	5.6	21.7	26
15	7.1	19.2	37
20	3.7	19.9	19
27	2.5	18.1	14
40	1.2	14.3	8.5
52	4.1	16.4	25
64	4.5	15.5	29
76	4.4	16.4	27

## Discussion

Table 2 summarizes the performance of CH_3_OH production in different types of MBRs with their operation conditions for comparison between the IMBR and others [33–36]. We designed the IMBR to reduce energy consumption for supplying gas in methanotrophic processes. In the IMBR, gas pressurizing is unnecessary, because cells on the membrane are placed in the gas phase. In the IMBR, the high CH_3_OH productivity was achieved, even though the gas flow rate was 0.2 mL min^−1^ into 2.5 mL of a reaction volume (gas chamber) (dilution rate *D* = 0.08 min^−1^), indicating low energy consumption for gas supply. The CH_3_OH productivity (0.74 mmol L^−1^ h^−1^) close to our data was achieved by Duan et al. using a different bacterial species than ours in a conventional MBR. However, in their paper, pure CH_4_ and O_2_ were supplied at the summed flow rate of 112 mL min^−1^ from long tubes into 300 mL reaction mixture (*D* = 0.37 min^−1^). To supply gas from tubes to the bulk water medium, a high pressure is usually required to overcome the transmembrane pressure. In addition, if we use pure oxygen, the CH_3_OH productivity will improve, because oxygen is stoichiometry short from mixed gas of 20% CH_4_ and 80% air supplied in this study; more than 20% O_2_ is required for stoichiometric CH_3_OH production from 20% CH_4_ and cell maintenance. As for conversion from CH_4_ into CH_3_OH, we cannot simply compare our values obtained by the continuous product discharge with the value obtained by product accumulation in a batch system by Duan et al*.* However, the maximum conversion in our batch operation was 60%, which was the similar level as the value shown by them. Of course, we should note that cell separation is unnecessary for purification of CH_3_OH in the IMBR, unlike most of other methods [33, 34, 36] in Table 2.

**Table 2:** Performance of CH_3_OH production in different types of membrane bioreactors

Refs.	Bacteria strain	Reactor type	Operation	Total production period (h)	MDH inhibition method	External reducing agent	CH_3_OH productivity (mmol L^−1^ h^−1^)	Conversion (%)
This study	*Methylococcus capsulatus* Bath	Inverse membrane bioreactor	Gas: continuous supply at 0.2 mL min^−1^ (CH_4_:air = 1: 4 *v*/*v*) Aqueous: continuous supply at 4 mL h^−1^ Reaction volume: 5 mL	76	10 μM Cyclopropanol	10 mM sodium formate	0.88	Max: 37^a^ Steady state: 27^b^
This study	*Methylococcus capsulatus* Bath	Inverse membrane bioreactor	Gas: continuous supply at 3 mL min^−1^ (CH_4_:air = 1: 4 *v*/*v*) Aqueous: batch Reaction volume: 5 mL	6	10 μM Cyclopropanol	10 mM sodium formate	0.62	Max: 60^c^ Overall: 24^d^
Duan et al. (2011) [34]	*Methylosinus trichosporium* OB3b	Bubble free membrane bioreactor	Gas: continuous supply at 112 mL min^−1^ (CH_4_:O_2_ = 1: 1 *v*/*v*) Aqueous: batch Reaction volume: 300 mL	40	400 mM phosphate 10 mM MgCl_2_	20 mM sodium formate	0.74	64
Pen et al. (2014) [33]	*Methylosinus trichosporium* OB3b	Batch membrane bioreactor	Gas: continuous supply at 94 mL min^−1^ (CH_4_:O_2_ = 1: 1 *v*/*v*) Aqueous: Batch Reaction volume: 50 mL	24	12.9 mM phosphate 100 mM NaCl 1.0 mM EDTA	20 mM sodium formate	0.38	N/D
Pen et al. (2016) [36]	*Methylosinus trichosporium* OB3b	Fed-batch membrane bioreactor	Gas: continuous supply at 94 mL min^−1^ (CH_4_:O_2_ = 1: 1 *v*/*v*) Aqueous: semi-batch Reaction volume: 150 mL	48	12.9 mM phosphate 100 mM NaCl 1.0 mM EDTA	20 mM sodium formate	8.15 × 10^–2^	N/D
Xin et al. (2004) [35]	*Methylosinus trichosporium* IMV 3011	Continuous stirred membrane reactor	Gas: continuous supply at 5 mL min^−1^ (CH_4_:O_2_:N_2_: CO_2_ = 1:1:1:2 *v*/*v*) Aqueous: continuous supply at 7 mL h^−1^ Reaction volume: 40 mL	198	Carbon dioxide	Partial CH_3_OH oxidation for NADH regeneration	2.90 × 10^–3^	N/D

^a^Data from the point at 15 h in Table 1

^b^Average of data from the points at 52 h, 64 h, and 76 h in Table 1

^c^Calculated from the data from the first 1 h operation of the experiment shown in Fig. 3b

^d^Calculated from the data from the 6 h operation of the experiment shown in Fig. 3b

The maximum consumption rate of CH_4_ was 58 μmol h^−1^ in a 2.5 mL gas chamber when *M. capsulatus* (Bath) resting cells of 10 mg-DCW were used without the addition of cyclopropanol and formate in the system (Additional file 3), resulting in the specific CH_4_ consumption rate of 5.8 mmol g-DCW^−1^ h^−1^, which is about 3 times lower than 18.46 mmol g-DCW^−1^ h^−1^ in literatures [37, 38]. This was probably because cells in a gas phase reaction are in a resting condition [39]. It is reasonable to deduce that resting cells should consume less carbon sources than actively growing cells for the production of single cell protein.

The diffusion coefficient of CH_4_ in air is much larger (0.22 cm^2^ s^−1^ at 25 °C, [40]) than that in water (0.15 × 10^–4^ cm^2^ s^−1^ at 25 °C, [41]). However, the results shown in Fig. 4c suggested that the external mass transfer, which refers to the process of delivering reactants from the bulk phase to catalysts on a solid surface, was limited in a flowing state in the gas chamber with a large size (25 mL). Then, the pattern of the gaseous flow in the gas chambers was simulated. This result predicts that channeling occurred in the gas flow; most CH_4_ molecules passed through without contacting the biocatalysts before being exhausted from the outlet (Additional file 7a, b). In contrast, it was simulated that the gas flow in the small chamber was distributed into a disk-like shape and CH_4_ molecules efficiently contacted the cells on a membrane (Additional file 7c, d). However, it is predicted that the velocity of the gas flow was slower in the small region of the opposite side of the inlet, because the outlet was located at the center of the disk-shaped gas chamber. Displacing the outlet to the opposite side of the inlet will improve the gas flow and spread the flow over the entire cells on a membrane. We also think the reactor can be scaled up while maintaining the same constant surface area-to-volume as the small chamber. Of course, the similar simulations are also useful when scaling up and/or modifying the configuration of IMBRs. Multiple IMBRs can also be stacked or arranged for further scale-up.

The concentration of cyclopropanol should be optimized, because it introduces a kind of trade-off relationship between the effectiveness for inhibiting the MDH activity and cell or MMO activity. When the cyclopropanol concentration provided to the aqueous solution increased from 0 μM, 1 μM, and 10 μM, the CH_4_ consumption ratio decreased from 11%, 10%, and 3.7%, respectively, while the conversion of CH_4_ into CH_3_OH increased from 0%, 4.6%, and 60%, respectively (Additional file 5). However, the slight decrease in the CH_4_ consumption ratio to 2.9% but the large decrease in the maximum conversion to 19% at 20 μM cyclopropanol suggested that a high concentration of this MDH inhibitor lowers the MMO activity (Additional file 6). Therefore, 10 μM was the optimum concentration of cyclopropanol for the conversion of CH_4_ into CH_3_OH under the experimental conditions for Fig. 6. The optimum concentration of cyclopropanol seems to depend on the amount of cells; the decrease in the CH_4_ conversion into CH_3_OH that was observed when the amount of the filtered cells increased from 12.5 mg-DCW to 25 mg-DCW at 10 μM cyclopropanol, as shown in Fig. 5b, suggests that this cyclopropanol concentration was not enough to effectively inhibit MDH in the increased cells.

In the experiment shown in Fig. 3, CH_3_OH concentration increased from 0 to 3.7 mM in 10 mL of the aqueous solution in 6 h, producing 37 μmol CH_3_OH. At the same time, the concentration of formate in 10 mL of the aqueous solution decreased from 10 to 3.7 mM in 6 h, consuming 63 μmol formate (Additional file 1). This implies that 63 μmol NADH was generated, in which 37 μmol of NADH was used for methanol production. Therefore, 60% of NADH generated from formate oxidation into CO_2_ contributed to methanol production, suggesting that 40% of the generated NADH was used for cell maintenance. It has also been reported in literatures that formate could be used by *Methylosinus trichosporium* OB3b in the serine pathway for carbon fixation, which would compete with the process of NADH generation [4, 36, 42]. The high productivity of CH_3_OH in the proposed IMBR was maintained over 76 h of the continuous operation, suggesting the long-term stability of the cells. The continuous supply of formate and cyclopropanol is also thought to be important for keeping the stable activity to avoid the lack of reducing power and the decrease in the inhibitory activity for MDH.

## Conclusion

The bioreactor constructed in this study, IMBR, combined not only the feature of the gas-phase bioreactor, which exhibits a low resistance to the gas delivery of CH_4_, but also that of MBRs, which supply chemicals and harvest CH_3_OH from the aqueous phase. Along with an efficient supply of gaseous substrates, MDH inhibitors, reducing agents, and nutrients, the production rate of CH_3_OH can be better enhanced using an inverse cake of the filtered cells in the IMBR compared to the conventional MBR. This design concept of the proposed bioreactor could inspire the development of a novel gas-phase bioreactor for converting various gaseous substrates, such as CH_4_, CO_2_ or syngas, into high-value liquid products.

## Methods

### Cultivation of Methylococcus capsulatus (Bath)

*M. capsulatus* (Bath) was grown in nitrate mineral salt (NMS) medium without the addition of copper ions for all experiments with CH_3_OH production or with 20 μM CuSO_4_ only for investigating the effect of the space time on the consumption rate of CH_4_ [43, 44]. *M. capsulatus* (Bath) cells were cultivated at 42 °C with a supplement of 20% (*v*/*v*) CH_4_ for 4 days. The cells were harvested by centrifugation at 6000 rpm for 30 min and resuspended in fresh NMS medium before experiments. The cell density was determined by the optical density at 540 nm. An optical density of 1 unit corresponded to approximately 0.225 g L^−1^ dry *M. capsulatus* (Bath) cells.

### Construction of membrane bioreactors

The components assembled in membrane bioreactors (MBRs) from top to bottom were as follows: a polycarbonate (PC) gas chamber with a volume of 2.5 mL or 25 mL, a polytetrafluoroethylene (PTFE) gasket, a membrane sheet with cells, a PC support grid, and a PC liquid chamber with a volume of 2.5 mL. These components were locked using screws and nuts to build up a gas-tight bioreactor [39].

An inverse membrane bioreactor (IMBR) was assembled with a hydrophilic glass fiber filter (GF/F 47; GE Healthcare) as a support for the filtered cells toward the gas phase. In a conventional membrane bioreactor (MBR), a hydrophobic polyvinylidene difluoride (PVDF) filter (GVHP04700; Durapore; Merck) was used to separate the gas phase and the aqueous phase.

### Cell preparation for methanol production

After harvesting *M. capsulatus* (Bath) by centrifugation, the cells were resuspended in 10 mL of fresh NMS medium with 10 μM cyclopropanol and incubated at room temperature for 60 min. After that, the cells treated with cyclopropanol were filtered as previously described [39] and then placed in the middle of the bioreactor. To investigate the effect of the space time and the effect of the cyclopropanol concentration, the cells were not treated with cyclopropanol in advance.

### Methanol production in a membrane bioreactor

The mixture of CH_4_ and air (80% N_2_ and 20% O_2_) was prepared by a gas blender (BR-2CS; KOFLOC Kyoto) to control the gas flow rate. Gas was introduced into the gas chamber continuously through a PTFE tube (0.5 m long × 3 mm O.D. × 2 mm I.D.). The aqueous solution that was composed of fresh NMR medium with cyclopropanol and sodium formate was circulated between the bottom liquid chamber and the solution container using a peristaltic pump (ISM931; ISMATEC) at 10 mL min^−1^. In the continuous operation of CH_3_OH production, in addition to the liquid circulation by a peristaltic pump, the same composition of the aqueous solution was injected into the bottom liquid chamber using a syringe pump (LEGATO 200; KD Scientific) at a liquid flow rate of 4 mL h^−1^ and continuously discharged into the solution container. The total volume of the aqueous solution circulated in the bioreactor was approximately 10 mL. To calculate CH_3_OH productivity, the reaction volume of the IMBR was defined at 5 mL, which was the summation of 2.5 mL of the gas chamber and 2.5 mL of the liquid chamber. The experiments were conducted at 42 °C.

### Quantification of methane consumption and methanol production

The concentrations of CH_4_ and CH_3_OH were determined quantitatively by injecting 0.1 mL of the gas sample and 5 μL of the aqueous sample, respectively, using a syringe into the injector port of a gas chromatograph (GC-2014; Shimadzu) equipped with a flame ionization detector and a 25% sorbitol Gasport B (60/80) glass column (GL Sciences). Gas chromatography parameters were nitrogen as the carrier gas at a flow rate of 40 mL min^−1^, column temperature of 100 °C, and detector temperature of 150 °C.

## Supplementary Information

**Additional file 1: **Time courses of the formate concentration, the CH_3_OH concentration, and pH in the aqueous solution in the experiment of Fig 3b

**Additional file 2: **The time course of OD values in the aqueous solution in the experiment of Fig 3b.

**Additional file 3: **Consumption rates and consumption ratios of CH_4_ calculated from the data shown in Fig 4c.

**Additional file 4: **Consumption ratios of CH_4_ and conversion calculated from the data shown in (a) Fig. 5a and (b) Fig. 5b.

**Additional file 5: **Consumption rates of CH_4_, consumption ratios of CH_4_, and conversion calculated from the data shown in Fig. 6a.

**Additional file 6: **Consumption rates of CH_4_, consumption ratios of CH_4_, and conversion calculated from the data shown in Fig. 6b.

**Additional file 7: **In a 25 mL gas chamber, (a) the profile of velocity magnitude on the XZ plane; and (b) the 3-D profile of the gas flow when the velocity magnitude is higher than 2 mm min^-1^. In a 2.5 mL gas chamber, (c) the profile of velocity magnitude on the XZ plane; (d) the 3-D profile of the gas flow when the velocity magnitude is higher than 2 mm min^-1^. The arrows are velocity vectors indicating the direction of the gas flow. The dashed circle indicates the region at a velocity slower than 2 mm min^-1^. The flow dynamics in the gas chamber were simulated by Autodesk CFD 2019. The material was set as an incompressible flow composed of 20% CH_4_ and 80% air at 101325 Pa and 315.15 K. The boundary conditions were a volume flow rate of 1 cm^3^ min^-1^ with a fully developed flow at the inlet and a gauge pressure of 0 Pa at the outlet. The mesh size of the model was set by automatic sizing with a minimum refinement length of 0.1 mm. The model was solved by the advection mode of ADV 1 (monotone streamline upwind) in a laminar state without heat transfer. The velocity magnitude is normalized in the range from 0 to 5 mm min^-1^. A velocity higher than 5 mm min^-1^ is included in red.

## Data Availability

The data sets used and/or analyzed during the current study are available from the corresponding author on reasonable request.

## Abbreviations

CH_4_: Methane
CH_3_OH: Methanol
CO_2_: Carbon dioxide
GTL: Gas-to-liquid
I.D.: Inner diameter
IMBR: Inverse membrane bioreactor
MBR: Membrane bioreactor
MMO: Methane monooxygenase
MDH: Methanol dehydrogenase
*M. capsulatus*: *Methylococcus capsulatus*
NMS: Nitrate mineral salt
N_2_: Nitrogen
O_2_: Oxygen
O.D.: Outer diameter
PC: Polycarbonate
PTFE: Polytetrafluoroethylene
PVDF: Polyvinylidene difluoride
