# Supplementary material for: A novel inverse membrane bioreactor for efficient bioconversion from methane gas to liquid methanol using a microbial gas-phase reaction
Source: Biotechnol Biofuels Bioprod. 2023 Feb 2;16:16. doi: 10.1186/s13068-023-02267-6 (PMC9893580; doi:10.1186/s13068-023-02267-6)
Supplement: Supplementary file 1 — Additional file 1: Time courses of the formate concentration, the CH3OH concentration, and pH in the aqueous solution in the experiment of Fig 3b [file 13068_2023_2267_MOESM1_ESM.docx]

Supplementary information

A novel inverse membrane bioreactor for efficient bioconversion from methane gas to liquid methanol using a microbial gas-phase reaction

Yan-Yu Chen^1^, Masahito Ishikawa^1^, Katsutoshi Hori^1,*^

^1^ Department of Biotechnology, Graduate School of Engineering, Nagoya University, Furo-cho, Chikusa-ku, Nagoya 464-8603, Japan.

*Corresponding authors: Katsutoshi Hori

Department of Biomolecular Engineering, Graduate School of Engineering, Nagoya University, Furo-cho, Chikusa-ku, Nagoya 464-8603, Japan

Tel.: +81-52-789-3339; Fax: +81-52-789-3218

E-mail address: [khori@chembio.nagoya-u.ac.jp](mailto:khori@chembio.nagoya-u.ac.jp)

Additional file 1. Time courses of the formate concentration, the CH_3_OH concentration, and pH in the aqueous solution in the experiment of Fig 3b.
