# Supplementary material for: A novel inverse membrane bioreactor for efficient bioconversion from methane gas to liquid methanol using a microbial gas-phase reaction
Source: Biotechnol Biofuels Bioprod. 2023 Feb 2;16:16. doi: 10.1186/s13068-023-02267-6 (PMC9893580; doi:10.1186/s13068-023-02267-6)
Supplement: Supplementary file 2 — Additional file 2: The time course of OD values in the aqueous solution in the experiment of Fig 3b. [file 13068_2023_2267_MOESM2_ESM.docx]

Supplementary information

A novel inverse membrane bioreactor for efficient bioconversion from methane gas to liquid methanol using a microbial gas-phase reaction

Yan-Yu Chen^1^, Masahito Ishikawa^1^, Katsutoshi Hori^1,*^

^1^ Department of Biotechnology, Graduate School of Engineering, Nagoya University, Furo-cho, Chikusa-ku, Nagoya 464-8603, Japan.

*Corresponding authors: Katsutoshi Hori

Department of Biomolecular Engineering, Graduate School of Engineering, Nagoya University, Furo-cho, Chikusa-ku, Nagoya 464-8603, Japan

Tel.: +81-52-789-3339; Fax: +81-52-789-3218

E-mail address: [khori@chembio.nagoya-u.ac.jp](mailto:khori@chembio.nagoya-u.ac.jp)

**Additional file 2**. The time course of OD values in the aqueous solution in the experiment of Fig 3b.

| Time points | 0 h | 1 h | 2 h | 3 h | 4 h | 5 h | 6 h |
| --- | --- | --- | --- | --- | --- | --- | --- |
| OD_540nm_ | 0.005 | 0.005 | 0.001 | 0.004 | 0.004 | 0.005 | 0.005 |
