# Supplementary material for: A novel inverse membrane bioreactor for efficient bioconversion from methane gas to liquid methanol using a microbial gas-phase reaction
Source: Biotechnol Biofuels Bioprod. 2023 Feb 2;16:16. doi: 10.1186/s13068-023-02267-6 (PMC9893580; doi:10.1186/s13068-023-02267-6)
Supplement: Supplementary file 3 — Additional file 3: Consumption rates and consumption ratios of CH4 calculated from the data shown in Fig 4c. [file 13068_2023_2267_MOESM3_ESM.docx]

Supplementary information

A novel inverse membrane bioreactor for efficient bioconversion from methane gas to liquid methanol using a microbial gas-phase reaction

Yan-Yu Chen^1^, Masahito Ishikawa^1^, Katsutoshi Hori^1,*^

^1^ Department of Biotechnology, Graduate School of Engineering, Nagoya University, Furo-cho, Chikusa-ku, Nagoya 464-8603, Japan.

*Corresponding authors: Katsutoshi Hori

Department of Biomolecular Engineering, Graduate School of Engineering, Nagoya University, Furo-cho, Chikusa-ku, Nagoya 464-8603, Japan

Tel.: +81-52-789-3339; Fax: +81-52-789-3218

E-mail address: [khori@chembio.nagoya-u.ac.jp](mailto:khori@chembio.nagoya-u.ac.jp)

**Additional file 3.** Consumption rates and consumption ratios of CH_4_ calculated from the data shown in Fig 4c.

|  | 2.5 mL of the gas chamber | | 25 mL of the gas chamber | |
| --- | --- | --- | --- | --- |
| Time point (h) | Consumption rate of CH_4_ (μmol h^-1^) | Consumption ratio of CH_4_ (%) | Consumption rate of CH_4_ (μmol h^-1^) | Consumption ratio of CH_4_ (%) |
| 0.25 | 26 | 5.7 | 1.4 | 0.3 |
| 0.5 | 47 | 10 | 1.4 | 0.3 |
| 1 | 55 | 12 | 1.8 | 0.4 |
| 1.5 | 58 | 13 | 2.4 | 0.5 |
| 2 | 57 | 12 | 2.4 | 0.5 |
| 2.5 | 58 | 12 | 2.3 | 0.5 |
| 3 | 58 | 13 | 2.3 | 0.5 |
