# Supplementary material for: A novel inverse membrane bioreactor for efficient bioconversion from methane gas to liquid methanol using a microbial gas-phase reaction
Source: Biotechnol Biofuels Bioprod. 2023 Feb 2;16:16. doi: 10.1186/s13068-023-02267-6 (PMC9893580; doi:10.1186/s13068-023-02267-6)
Supplement: Supplementary file 4 — Additional file 4: Consumption ratios of CH4 and conversion calculated from the data shown in (a) Fig. 5a and (b) Fig. 5b. [file 13068_2023_2267_MOESM4_ESM.docx]

Supplementary information

A novel inverse membrane bioreactor for efficient bioconversion from methane gas to liquid methanol using a microbial gas-phase reaction

Yan-Yu Chen^1^, Masahito Ishikawa^1^, Katsutoshi Hori^1,*^

^1^ Department of Biotechnology, Graduate School of Engineering, Nagoya University, Furo-cho, Chikusa-ku, Nagoya 464-8603, Japan.

*Corresponding authors: Katsutoshi Hori

Department of Biomolecular Engineering, Graduate School of Engineering, Nagoya University, Furo-cho, Chikusa-ku, Nagoya 464-8603, Japan

Tel.: +81-52-789-3339; Fax: +81-52-789-3218

E-mail address: [khori@chembio.nagoya-u.ac.jp](mailto:khori@chembio.nagoya-u.ac.jp)

**Additional file 4.** Consumption ratios of CH_4_ and conversion calculated from the data shown in (a) Fig 5a and (b) Fig 5b.

(a)

| CH_4_ inlet concentration (% (v/v)) | Consumption ratio of CH_4_ (%) | Conversion (%) |
| --- | --- | --- |
| 2 | 0.7 | 37 |
| 5 | 1.3 | 48 |
| 10 | 2.8 | 57 |
| 20 | 3.7 | 59 |
| 30 | 3.7 | 60 |

(b)

| Immobilized cell amount (mg per bioreactor) | Consumption ratio of CH_4_ (%) | Conversion (%) |
| --- | --- | --- |
| 6.25 | 1.4 | 58 |
| 12.5 | 3.7 | 58 |
| 25 | 4.5 | 33 |
| 50 | 4.9 | 27 |
| 100 | 4.6 | 26 |
