# Supplementary material for: A novel inverse membrane bioreactor for efficient bioconversion from methane gas to liquid methanol using a microbial gas-phase reaction
Source: Biotechnol Biofuels Bioprod. 2023 Feb 2;16:16. doi: 10.1186/s13068-023-02267-6 (PMC9893580; doi:10.1186/s13068-023-02267-6)
Supplement: Supplementary file 5 — Additional file 5: Consumption rates of CH4, consumption ratios of CH4, and conversion calculated from the data shown in Fig. 6a. [file 13068_2023_2267_MOESM5_ESM.docx]

Supplementary information

A novel inverse membrane bioreactor for efficient bioconversion from methane gas to liquid methanol using a microbial gas-phase reaction

Yan-Yu Chen^1^, Masahito Ishikawa^1^, Katsutoshi Hori^1,*^

^1^ Department of Biotechnology, Graduate School of Engineering, Nagoya University, Furo-cho, Chikusa-ku, Nagoya 464-8603, Japan.

*Corresponding authors: Katsutoshi Hori

Department of Biomolecular Engineering, Graduate School of Engineering, Nagoya University, Furo-cho, Chikusa-ku, Nagoya 464-8603, Japan

Tel.: +81-52-789-3339; Fax: +81-52-789-3218

E-mail address: [khori@chembio.nagoya-u.ac.jp](mailto:khori@chembio.nagoya-u.ac.jp)

**Additional file 5.** Consumption rates of CH_4_, consumption ratios of CH_4_, and conversion calculated from the data shown in Fig 6a.

| Solution compositions | Time point (h) | Consumption rate of CH_4_ (μmol h^-1^) | Consumption ratio of CH_4_ (%) | Conversion (%) |
| --- | --- | --- | --- | --- |
| 10 mM Formate  + 0 μM Cyclopropanol | 0 | --- | --- | --- |
|  | 0.5 | 54 | 12 |  |
|  | 1 | 55 | 12 | --- |
|  | 1.5 | 49 | 11 |  |
|  | 2 | 51 | 11 | --- |
|  | 2.5 | 53 | 11 |  |
|  | 3 | 50 | 11 | --- |
|  |  |  |  |  |
| 10 mM Formate  + 1 μM Cyclopropanol |  |  |  |  |
|  | 3.5 | 47 | 10 |  |
|  | 4 | 44 | 9.5 | 4.6 |
|  | 4.5 | 46 | 10 |  |
|  | 5 | 52 | 11 | 2.2 |
|  | 5.5 | 48 | 10 |  |
|  | 6 | 48 | 10 | 1.6 |
|  |  |  |  |  |
| 10 mM Formate  + 10 μM Cyclopropanol |  |  |  |  |
|  | 6.5 | 16 | 3.5 |  |
|  | 7 | 17 | 3.6 | 60 |
|  | 7.5 | 18 | 4.0 |  |
|  | 8 | 16 | 3.5 | 43 |
|  | 8.5 | 17 | 3.8 |  |
|  | 9 | 18 | 3.9 | 21 |
