# Supplementary material for: A novel inverse membrane bioreactor for efficient bioconversion from methane gas to liquid methanol using a microbial gas-phase reaction
Source: Biotechnol Biofuels Bioprod. 2023 Feb 2;16:16. doi: 10.1186/s13068-023-02267-6 (PMC9893580; doi:10.1186/s13068-023-02267-6)
Supplement: Supplementary file 6 — Additional file 6: Consumption rates of CH4, consumption ratios of CH4, and conversion calculated from the data shown in Fig. 6b. [file 13068_2023_2267_MOESM6_ESM.docx]

Supplementary information

A novel inverse membrane bioreactor for efficient bioconversion from methane gas to liquid methanol using a microbial gas-phase reaction

Yan-Yu Chen^1^, Masahito Ishikawa^1^, Katsutoshi Hori^1,*^

^1^ Department of Biotechnology, Graduate School of Engineering, Nagoya University, Furo-cho, Chikusa-ku, Nagoya 464-8603, Japan.

*Corresponding authors: Katsutoshi Hori

Department of Biomolecular Engineering, Graduate School of Engineering, Nagoya University, Furo-cho, Chikusa-ku, Nagoya 464-8603, Japan

Tel.: +81-52-789-3339; Fax: +81-52-789-3218

E-mail address: [khori@chembio.nagoya-u.ac.jp](mailto:khori@chembio.nagoya-u.ac.jp)

**Additional file 6.** Consumption rates of CH_4_, consumption ratios of CH_4_, and conversion calculated from the data shown in Fig 6b.

| Solution compositions | Time point (h) | Consumption rate of CH_4_ (μmol h^-1^) | Consumption ratio of CH_4_ (%) | Conversion (%) |
| --- | --- | --- | --- | --- |
| 10 mM Formate  + 20 μM Cyclopropanol | 0 | --- | --- | 0 |
|  | 1 | 13 | 2.8 | 7.5 |
|  | 3 | 14 | 3.0 | 19 |
|  | 4 | 15 | 3.2 | 12 |
|  | 5 | 14 | 3.1 | 9.6 |
|  | 6 | 13 | 2.8 | 6.1 |
|  | 7 | 13 | 2.8 | 2.1 |
