# Supplementary material for: A novel inverse membrane bioreactor for efficient bioconversion from methane gas to liquid methanol using a microbial gas-phase reaction
Source: Biotechnol Biofuels Bioprod. 2023 Feb 2;16:16. doi: 10.1186/s13068-023-02267-6 (PMC9893580; doi:10.1186/s13068-023-02267-6)
Supplement: Supplementary file 7 — Additional file 7: In a 25 mL gas chamber, (a) the profile of velocity magnitude on the XZ plane; and (b) the 3-D profile of the gas flow when the velocity magnitude is higher than 2 mm min-1. In a 2.5 mL gas chamber, (c) the profile of velocity magnitude on the XZ plane; (d) the 3-D profile of the gas flow when the velocity magnitude is higher than 2 mm min-1. The arrows are velocity vectors indicating the direction of the gas flow. The dashed circle indicates the region at a velocity slower than 2 mm min-1. The flow dynamics in the gas chamber were simulated by Autodesk CFD 2019. The material was set as an incompressible flow composed of 20% CH4 and 80% air at 101325 Pa and 315.15 K. The boundary conditions were a volume flow rate of 1 cm3 min-1 with a fully developed flow at the inlet and a gauge pressure of 0 Pa at the outlet. The mesh size of the model was set by automatic sizing with a minimum refinement length of 0.1 mm. The model was solved by the advection mode of ADV 1 (monotone streamline upwind) in a laminar state without heat transfer. The velocity magnitude is normalized in the range from 0 to 5 mm min-1. A velocity higher than 5 mm min-1 is included in red. [file 13068_2023_2267_MOESM7_ESM.docx]

Supplementary information

A novel inverse membrane bioreactor for efficient bioconversion from methane gas to liquid methanol using a microbial gas-phase reaction

Yan-Yu Chen^1^, Masahito Ishikawa^1^, Katsutoshi Hori^1,*^

^1^ Department of Biotechnology, Graduate School of Engineering, Nagoya University, Furo-cho, Chikusa-ku, Nagoya 464-8603, Japan.

*Corresponding authors: Katsutoshi Hori

Department of Biomolecular Engineering, Graduate School of Engineering, Nagoya University, Furo-cho, Chikusa-ku, Nagoya 464-8603, Japan

Tel.: +81-52-789-3339; Fax: +81-52-789-3218

E-mail address: [khori@chembio.nagoya-u.ac.jp](mailto:khori@chembio.nagoya-u.ac.jp)

**Additional file 7.** In a 25 mL gas chamber, (a) the profile of velocity magnitude on the XZ plane; and (b) the 3-D profile of the gas flow when the velocity magnitude is higher than 2 mm min^-1^. In a 2.5 mL gas chamber, (c) the profile of velocity magnitude on the XZ plane; (d) the 3-D profile of the gas flow when the velocity magnitude is higher than 2 mm min^-1^. The arrows are velocity vectors indicating the direction of the gas flow. The dashed circle indicates the region at a velocity slower than 2 mm min^-1^. The flow dynamics in the gas chamber were simulated by Autodesk CFD 2019. The material was set as an incompressible flow composed of 20% CH_4_ and 80% air at 101325 Pa and 315.15 K. The boundary conditions were a volume flow rate of 1 cm^3^ min^-1^ with a fully developed flow at the inlet and a gauge pressure of 0 Pa at the outlet. The mesh size of the model was set by automatic sizing with a minimum refinement length of 0.1 mm. The model was solved by the advection mode of ADV 1 (monotone streamline upwind) in a laminar state without heat transfer. The velocity magnitude is normalized in the range from 0 to 5 mm min^-1^. A velocity higher than 5 mm min^-1^ is included in red.
